# Exploiting macropinocytosis for drug delivery into KRAS mutant cancer

**DOI:** 10.7150/thno.67889

**Published:** 2022-01-01

**Authors:** Huiqin Liu, Feng Qian

**Affiliations:** School of Pharmaceutical Sciences, Beijing Advanced Innovation Center for Structural Biology, and Key Laboratory of Bioorganic Phosphorus Chemistry & Chemical Biology (Ministry of Education), Tsinghua University, Beijing 100084, P.R. China.

**Keywords:** Macropinocytosis, KRAS, drug delivery, pancreatic cancer

## Abstract

KRAS mutations are one of the most common gene mutations linked to cancer, presenting in approximately 25% of all tumors, especially pancreatic, lung, and colorectal cancers. Mutant KRAS has long been considered an undruggable target, stalling progress in direct KRAS targeting for many years, while targeted drug delivery into KRAS mutant cells utilizing their transformed metabolic behavior might present an alternative opportunity. Macropinocytosis, a nonselective, fluid-phase, endocytic route, was found to be upregulated as a metabolic feature in KRAS-driven tumors and plays a critical role in nutrient acquisition from extracellular fluids. With the observation that a variety of drug delivery systems could be internalized by KRAS mutant cancer cells through macropinocytosis, exploiting macropinocytosis for intracellular delivery of therapeutics into KRAS mutant tumor cells is emerging as a new drug delivery expedition. In this article, we summarized cancer biology studies that examined KRAS mutation-induced macropinocytosis, reviewed recent studies exploiting macropinocytosis enhancement for KRAS mutant cancer cell-selective drug delivery, and discussed the potential opportunities, challenges and pitfalls of this strategy.

## Introduction

Active transportation of extracellular substances into cells is one of the basic activities of living cells and is required to implement many important biological functions, such as signal transduction, nutrient absorption, and antigen uptake 1. The major endocytic routes, including macropinocytosis, clathrin-mediated endocytosis, caveolae-mediated endocytosis, and clathrin- or caveolae-independent endocytosis, are characterized by diverse regulation mechanisms and endocytic vesicles with different sizes and biological natures 1. Among them, macropinocytosis is a receptor-independent, actin-dependent, nonselective endocytic pathway utilized by cells to “drink” fluids and solutes from the external milieu, which was defined based on morphological observation in the early days 2. As early as 1931, Warren Lewis first described this process morphologically. Upon growth factor stimulation, waving sheet-like extensions of the plasma membrane were observed in rat macrophages, and evolved into heterogeneous phase-bright organelles with a diameter greater than 0.2 μm 3. To distinguish this pathway from endocytic processes involving smaller vesicles, Lewis named this activity pinocytosis (later renamed macropinocytosis), and the vesicles generated in this pathway macropinosome. Later, macropinocytosis, but not other receptor-mediated endocytosis pathways, was found to be selectively inhibited by amiloride, a Na^+^/H^+^ exchangers (NHE) inhibitor 4. The functional association between NHE remained unknown for many years until Koivusalo et al. addressed mechanisms mediating NHE inhibition of EGF-induced macropinocytosis in 2010 5. They confirmed that pH maintenance, rather than NHE activity itself or the associated Na^+^ gain, is required for macropinocytosis in A431 cells. Briefly, upon NHE inhibition, H^+^ extrusion was impaired, resulting in decreased cytosolic pH, which exclusively impaired the recruitment and activation of Rac1 and Cdc42 to membrane ruffles. Subsequently, suppression with amiloride and its analog 5-(NEthyl-N-isopropyl) amiloride (EIPA) has been widely used to identify macropinocytosis 6. To quantify the intensity of macropinocytosis in certain cell types, detecting the uptake of fluorescence-labelled agents, such as dextran, a known macropinocytosis marker, is the most common and usually also the only option 7.

The formation, trafficking and maturation of macropinosomes through the endocytic system and the regulatory mechanisms responsible for these dynamic processes have been comprehensively reviewed in several other excellent reviews 6,8,9,10 and thus will not be repeated here. Briefly, with the activation of small GTPases (such as Rac1) and the cell division control protein 42 homolog (Cdc42), the macropinocytosis process begins. Actin-rich, sheet-like membrane protrusions or ruffles are produced and further form circular cups. Enclosing these cups into macropinosomes depends on the production of phosphatidylinositol (3,4,5)-triphosphate (PIP3) and the inactivation of Rac1. The newly generated macropinosomes can fuse with the plasma membrane and release their contents back to the extracellular space or be transported to the lysosome for degradation.

Macropinocytosis is considered to be a conserved endocytic pathway shared by all vertebrate cells, while the regulation and functions of macropinocytosis in different cells could differ, as reviewed elsewhere 2,11. A molecular-level understanding of macropinocytosis and its signalling networks has gradually been unveiled 12,13. Generally, macropinocytosis in normal vertebrate cells is induced in response to growth factor stimulation (EGF, PDGF, etc.); however, some special cell types, such as innate immune cells and KRAS-transformed cancer cells, have intrinsic constitutive macropinocytosis apart from the induced forms 14. In the sight of immunology, macropinocytosis of dendritic cells and macrophages have been most studied, which is a major pathway for the capture of antigens 11. In KRAS-transformed cancer cells, enhanced macropinocytosis was recognized as a metabolic adaptation under nutrient stress conditions, supporting the survival and proliferation of aggressive tumors by scavenging extracellular proteins, lipids and cell debris 12,15,16.

KRAS is an oncogene protein with the capability of controlling diverse cellular functions, including macropinocytosis 17,12. Wild-type KRAS signalling is well controlled by switching between the active GTP-bound state and inactive GDP-bound state, stimulated by upstream EGFR activation and deactivated by guanine-nucleotide exchange factors 18. KRAS mutations, most of which (84%) occur at G12, resulting in single amino acid substitutions that activate the oncoprotein by hindering its ability to hydrolyze GTP, were detected in various solid tumors, such as lung cancer (~25% of cases), colorectal cancer (~35% of cases), and pancreatic cancer (~95% of cases), affecting an estimated 147,000 patients per year in the US 19,20. Although sotorasib (Lumakras^TM^), an inhibitor specifically targeting glycine to cysteine (G12C) missense mutations has been approved by US FDA, to date, there are arduous challenges to develop molecular targeted therapies against other KRAS mutations due to the special protein structure and biochemical properties of KRAS, making the strategy of targeting drug delivery into KRAS mutant cancer cells an attractive alternate 21.

The supposition that KRAS mutant tumor cells have constitutive and enhanced macropinocytosis drives an interesting question: could such enhanced macropinocytosis be distinctive enough to act as a *“phenotypic target”* for KRAS mutant cancer, where targeted therapeutics are urgently needed? Apparently, the addiction and dependence of KRAS mutant tumor cells on macropinocytosis lay the theoretical basis for obtaining therapeutic benefits by inhibiting macropinocytosis. On the other hand, instead of blocking macropinocytosis directly, some scientists are attempting to utilize this process to obtain drug delivery benefits. It has been reported that various drug delivery systems, including albumin, micelles, liposomes, exosomes, etc*.*, could be actively internalized by KRAS mutant cancer cells through macropinocytosis, suggesting that macropinocytosis regulation in the tumor setting can be harnessed for the delivery of anticancer therapeutics. In this article, we focused on constitutive macropinocytosis in KRAS mutant cancer cells and discussed the significance of this biological process from a drug delivery perspective. We analyzed the specificity and robustness of this phenomenon as a drug target candidate, summarized recent efforts in exploiting macropinocytosis for drug delivery into KRAS mutant cancer, and discussed the opportunities, challenges and pitfalls of this strategy.

## KRAS-induced macropinocytosis

One of the distinctive features that define macropinocytosis is the fact that it can be stimulated by growth factors, including EGF. KRAS is downstream of the EGFR signalling pathway, could be activated by either growth factor stimulation or oncogenic mutation, leading to the stimulation of different signal transduction pathways, including Rac, Cdc42, etc., that are necessary for macropinocytosis. The mechanistic underpinnings of oncogenic KRAS-induced macropinocytosis have been reviewed elsewhere 12. Here, we focus on the literature and studies that define the correlation and causality between oncogenic KRAS and macropinocytosis.

There are three closely related RAS isoforms: HRAS, KRAS, and NRAS in mammalian cells playing a central role in the regulation of cell growth and differentiation. Oncogenic HRAS protein (rather than gene)-induced macropinocytosis was reported as early as 1986 by Bar-Sagi and Feramisco, when RAS oncogenes were considered to be one of the contributing events of certain types of human cancers 22. To determine the cell activity directly triggered by the RAS protein, human HRAS protein was transformed into quiescent rat embryonic fibroblasts (REF-52) via microinjection. After injection, cell surface ruffles were induced within 30 mins to 1 hour, as revealed by scanning electron microscopy, and fluid-phase pinocytosis was significantly increased, as measured by uptake of fluorescein-conjugated dextran. Both the proto-oncogenic HRAS protein and oncogenic HRAS protein can induce rapid enhancement of cell membrane ruffles and pinocytosis. The difference is that the effect of oncogenic protein is relatively long-acting for more than 15 hours after injection, while the effect of proto-oncogenic protein is short-lived, limited to an interval of 3 hours after injection. Dose dependence of the stimulation of pinocytosis by the HRAS oncogene protein was also demonstrated in this study. The abilities of the HRAS and KRAS isoforms to induce membrane ruffling and macropinocytosis was compared in a follow-up study by the Bar-Sagi group 23. The average surface area of KRAS^G12V^-induced membrane ruffles and the number of KRAS^G12V^-induced pinocytic vesicles per cell were found to be ~2-fold greater than those of the HRAS^G12V^ groups, suggesting a stronger capability of macropinocytosis induction by KRAS^G12V^, since the expression levels and cellular distribution of the two were similar. To the best of our knowledge, there have not been published researches regarding NRAS related macropinocytosis.

## Robustness of macropinocytosis in KRAS mutant cancer cells

It is worth first commenting on the robustness of elevated macropinocytosis of KRAS mutant cancer cells because any conclusion that is true but only true under an extremely narrow set of conditions is therapeutically intractable regardless of effect size 24. In other words, we need to confirm that elevated macropinocytosis of KRAS mutant cancer cells could be reproduced consistently over a range of experimental conditions (for example, by investigating multiple cell lines or patient tumor samples in the setting of different KRAS mutant alleles, reproducing the findings in independent studies).

KRAS mutation is an oncogenic driver in solid tumors, including but not limited to pancreatic cancer, where KRAS mutation-induced macropinocytosis has been studied relatively earlier and more comprehensively. A study published in 2013 by Commisso and colleagues demonstrated that macropinocytosis of proteins in RAS-transformed cells acting as an amino acid supply route 16. Briefly, robust levels of macropinocytosis have been demonstrated in PDAC cell lines that harbor oncogenic KRAS mutations, observed in tumors of KPC mice (LSL-KRAS^G12D^; TP53^R172H/+^; Pdx1-Cre; a transgenic spontaneous pancreatic cancer mouse model), and meaningfully, detected in human PDAC tumor samples regardless of some intratumoral variability. The necessity and sufficiency of KRAS mutation for macropinocytosis induction have been verified using classical *in vitro* experimental systems by KRAS knockdown in cell lines with KRAS mutation and transforming exogenous KRAS mutation into normal cells like NIH3T3. The macropinocytosis levels in these experiments were detected under similar experimental settings, that is, cells or tissue samples were incubated in a serum-free medium containing 1-2 mg/mL fluorescently labelled dextran for 30 minutes and analyzed by fluorescence microscopy. We have assessed whether this biological phenomenon was robust and persistent enough to be exploited for drug delivery in a previous study, wherein we compared the uptake kinetics of FITC-dextran (~70 kDa) by KRAS-mutant (mt) and KRAS wild-type (wt) cells across wide ranges of concentrations and times using fluorescence-activated cell sorting (FACS) rather than microscope 25. Although the methods and experimental conditions are different, the trends are similar: MIA PaCa-2, which is a human pancreatic cell line with mutant KRAS, demonstrated obviously elevated macropinocytosis compared to the KRAS wt pancreatic cancer cell line BxPC-3. Across a period of 0.5-8 h and a dextran concentration range from 0.2-5 mg/mL, elevated extents and kinetics of macropinocytosis were also observed in KRAS^G12V^ transformed BxPC-3 cells compared to BxPC-3 control cells.

KRAS mutations are a cluster containing different versions, and several KRAS mutant subtypes related to the effect of macropinocytosis have been studied, including G12C, G12D, G12V, and G12R. Simply put, the first three subtypes have shown the capability to induce macropinocytosis in different studies, with the exception of G12R. MIA PaCa-2, a human pancreatic cancer cell line with a homozygous KRAS^G12C^ allele, displayed appreciably higher macropinocytosis levels as measured by 70 kDa dextran uptake compared to another pancreatic cell line with wild-type KRAS (i.e. BxPC-3) 16,25. Macropinosomes were detected in pancreatic intraepithelial neoplasia lesions derived from KPC mice harboring KRAS^G12D^, while pancreases collected from wild-type mice were found to be negative for macropinocytosis signals. Oncogenic KRAS^GV12^ was also proven to be sufficient to stimulate robust EIPA-sensitive TMR-dextran uptake in mouse NIH 3T3 cells and BxPC-3 cells 16,25. In agreement with these findings, Aaron Hobbs *et al.* found that transient siRNA suppression of mutant KRAS reduced macropinocytosis in KRAS^G12D^, KRAS^G12V^, and KRAS^G12C^ mutant cell lines in a separate study 20. The surprising discovery of this research is that atypical KRAS mutation G12R, which is relatively rare (~1% in lung and colorectal cancers, ~20% in PDAC), has unique properties in driving macropinocytosis. Suppression of KRAS did not reduce macropinocytosis in any of the seven KRAS^G12R^ mutant pancreatic cancer cell lines investigated in their study, and KRAS^G12R^ uniquely failed to stimulate macropinocytosis in three model systems (rat intestinal epithelial cells RIE-1, mouse fibroblasts NIH/3T3 and hTERT-immortalized human pancreatic duct-derived epithelial cells), similar to KRAS^G12D^ or KRAS^G12V^ 20. In terms of mechanism, KRAS^G12R^ is impaired in PI3K-AKT activation, which is required for macropinocytosis in KRAS^G12R^-mutant PDAC, due to structural perturbations and thus defective for interaction with p110α PI3K, a key downstream effector of KRAS.

The pervasiveness of macropinocytosis in various malignant tumors has been summarized by Commisso and colleagues 26. Since their initial study was published in 2013, which focused on KRAS-mutated pancreatic cancer, macropinocytosis has been recognized as a prevalent metabolic feature of other solid tumors, including lung, prostate, bladder and colon cancer, demonstrated by accumulating studies employing different *in vitro* systems as well as *in vivo* animal models. For example, Qian and colleagues explored the role of macropinocytosis in A549, a human non-small cell lung cancer (NSCLC) cell line that is addicted to oncogenic KRAS. Relying on integrin avβ3 and its modulator galectin-3, A549 NSCLC cells exhibited high levels of macropinocytosis to sustain their cellular fitness 27. Nevertheless, it has become apparent that the driving forces for cancer-associated macropinocytosis may be more diversified than expected since independent studies revealed that macropinocytosis could be enhanced by KRAS, HRAS, EGFR activation 28, galectin-3 27, loss of PTEN 29, Src activation 30, etc.

## The difference in macropinocytosis in KRAS mt cancer cell vs. KRAS wt cells

Is there a therapeutic window for macropinocytosis-based treatments against KRAS mutant cancer? A major challenge for such a treatment strategy is that macropinocytosis is a universal cellular process that exists in both KRAS mt cancer cells and normal cells. Therefore, a sufficiently distinctive macropinocytosis activity between cancer and normal cells is the significant determinant of the rationale for this strategy from the biological perspective, and quantitative analysis of the levels of macropinocytosis in KRAS mt tumor cells and KRAS wt normal cells can help to determine to what extent this KRAS-enhanced macropinocytosis can be harnessed for the delivery of anti-cancer therapeutics.

To date, the majority of the literature reports macropinocytosis levels measured by the method reported by Commisso et al., that is, incubating the cell or tissue sample in serum-free medium containing 1-2 mg/mL fluorescently labelled dextran for 30 minutes and counting the number of fluorescent spots per cell using a fluorescence microscope. Although elevated macropinocytosis of KRAS mt tumor cells has been repeatedly verified by different laboratories, the levels are largely variable, and there is currently no conclusive answer to the exact extent of macropinocytosis enhancement by mutant KRAS. Two reasons could have caused such complications. First, different experimental settings (timepoints, with/without serum depletion), analytical methods (macroscope or FACS) and different model systems (cell lines or tissues) could provide different results, and the difference could span two orders of magnitude (Table 1). Second, most of these studies used immortalized cells with wt KRAS as a control, such as NIH3T3, BxPC-3, and MCF-7, none of which are truly normal human cells.

We noticed that, in most of these studies, macropinocytosis levels in KRAS mutant cells and control cells were determined at a single time point, i.e., 30 min, with a few exceptions. The macropinocytosis flux of KRAS mutant cancer cells may be underestimated since induction macropinocytosis of oncogenic RAS protein was found to last for more than 15 hours, while endogenously expressed mutant proteins are expected to induce persistent macropinocytosis 22. It may provide extra useful information if the total amount of macropinocytosis flux across a certain time frame was calculated.

## Drug delivery systems exploiting macropinocytosis of KRAS mutant cancer cells

With these observations, exploiting macropinocytosis for intracellular drug delivery into KRAS mutant cancer is emerging as a recent exploration. Based on the uptake inhibition induced by EIPA (a Na^+^/H^+^ exchange blocker used as a specific inhibitor of macropinocytosis), a variety of delivery systems have been identified, of which macropinocytosis is the dominant internalization mechanism in KRAS mt cancer cells. As previously reviewed by Desai and colleagues, lipids, polymers, peptides, extracellular vesicles, and inorganic nanoparticles have been reported to be internalized by KRAS mt cancer cells through macropinocytosis 34.

Human serum albumin (HSA), the most abundant serum protein in the human body, has attracted much attention since it was reported that pancreatic cancer cells with KRAS mutations can take up extracellular albumin through macropinocytosis and degrade it into amino acids, serving as an important nutrient source to fuel their metabolic addiction. Based on this finding, we covalently conjugated the model drug doxorubicin to native albumin to imitate the endocytosis and intracellular trafficking of albumin to expand the therapeutic windows of the cytotoxic payload in KRAS mt PDAC utilizing enhanced macropinocytosis 25. We observed that doxorubicin-albumin conjugates exhibited EIPA-sensitive macropinocytosis in KRAS^G12V^-overexpressing BxPC-3 cells and MIA PaCa-2 cells with an inherent KRAS mutation but not in BxPC-3 control cells, in a similar manner as native albumin.

Abraxane^TM^ is a nanoparticle albumin-bound formulation of paclitaxel (referred to as nab-PTX), that is currently used as the first-line treatment for advanced pancreatic cancer in combination with gemcitabine. Macropinocytotic uptake of nab-PTX by KP1.9 cells that are derived from the tumor of lung adenocarcinoma mouse model and express KRAS^G12D^, was supported by EIPA inhibition and punctate subcellular colocalization of nab-PTX with fluorescent dextran 35. Consistent with previous reports regarding native albumin, transient KRAS^G12D^ overexpression in KRAS wt BxPC-3 cells and inducible KRAS^G12D^ expression in tumor cells derived from a genetically engineered mouse model of pancreatic cancer, both enhanced nab-PTX uptake by ≥8-fold. In addition to monomeric albumin, cross-linked albumin nanoparticles also demonstrated enhanced macropinocytotic uptake in cells with mutant RAS compared to RAS wild-type control cells, as reported by Liu *et al.* 36.

Albumin-based drug carriers have also been used to deliver protein therapeutics. Du and colleagues utilized HSA to deliver human β-defensin-2 (DF), a small cationic peptide that has antibacterial and antiviral activity and tumor cell cytotoxicity against MIA PaCa-2 cells and xenograft tumors 37. Based on the observation of fluorescence colocalization with dextran and the response to EIPA inhibition, the authors concluded that the internalization of DF-HSA in MIAPaCa-2 is apparently mediated by macropinocytosis and that there exists a differential pattern of intensity between KRAS mt MIAPaCa-2 cells and wt BxPC-3 cells. Wang et al. constructed the recombinant protein Fv-LDP-D3 by fusion domain III of HSA with an Fv fragment of an anti-EGFR antibody and the apoprotein of the antitumor antibiotic lidamycin (LDP) 38. The authors demonstrated that, with EGFR-targeting and macropinocytosis-intensifying bifunctional attributes, Fv-LDP-D3 has shown the remarkable potential of tumor imaging and prominent tumor inhibition in AsPC-1 (human pancreatic cancer cells with a homozygous KRAS^G12D^ mutation)-derived xenografts. The uptake mechanism of Fv-LDP-D3 in MIA PaCa-2 and AsPC-1 cells was identified by EIPA inhibition assay and colocalization of fluorescent macropinocytosis marker (TMR-dextran) and FITC-labeled recombinant proteins.

Exosomes are nanosized extracellular vesicles (40-150 nm) with a membrane lipid bilayer. They could be released by all cells and have been used to deliver siRNA to specifically target KRAS^G12D^ in pancreatic tumors by Kamerkar and colleagues 32. They found that exosome uptake but not liposome uptake in PANC-1 cells (PDAC cell line with KRAS^G12D^) was reduced upon EIPA inhibition, suggesting that exosomes enter PANC-1 cells via macropinocytosis. Inspired by the application of exosomes in KRAS mutant pancreatic cancer treatment, Deng and colleagues constructed an exosome-mimicking membrane hybrid nanoplatform by fusing PEGylated lipids with the DC2.4 cell membrane for the benefits of easier generation and better loading efficiency 39. The uptake of these exosome-mimicking nanoparticles in KRAS mt PANC-1 cells was significantly higher than that in BxPC-3 cells and was significantly decreased in response to EIPA inhibition, suggesting that this biomimetic system also imitates the uptake pattern of exosomes.

Dextran, a bacterial polysaccharide constructed by α-(1-6)-linked D-glucose units with different ratios of linkages and branches, has been used in drug delivery in the past to achieve solubilization or systemic PK modification before it became a popular “macropinocytosis marker” 40,41. Based on the hypothesis that coupling anti-cancer drugs to high-molecular-weight carriers may optimize drug disposition and improve tumor inhibition efficacy, a doxorubicin-dextran (70 kDa) conjugate was synthesized via Schiff's base reaction, exhibited better antitumor activity and optimized biodistribution compared to the free drug in tumor models and was evaluated in a clinical phase I trial for patients with refractory solid tumors in 1993 42,43. Recently, we also confirmed the feasibility of selectively targeting KRAS mutant pancreatic cancer with 70 kDa dextran-drug conjugates via both *in vitro* cellular studies and *in vivo* tumor model assessment 44.

**Figure 1:**
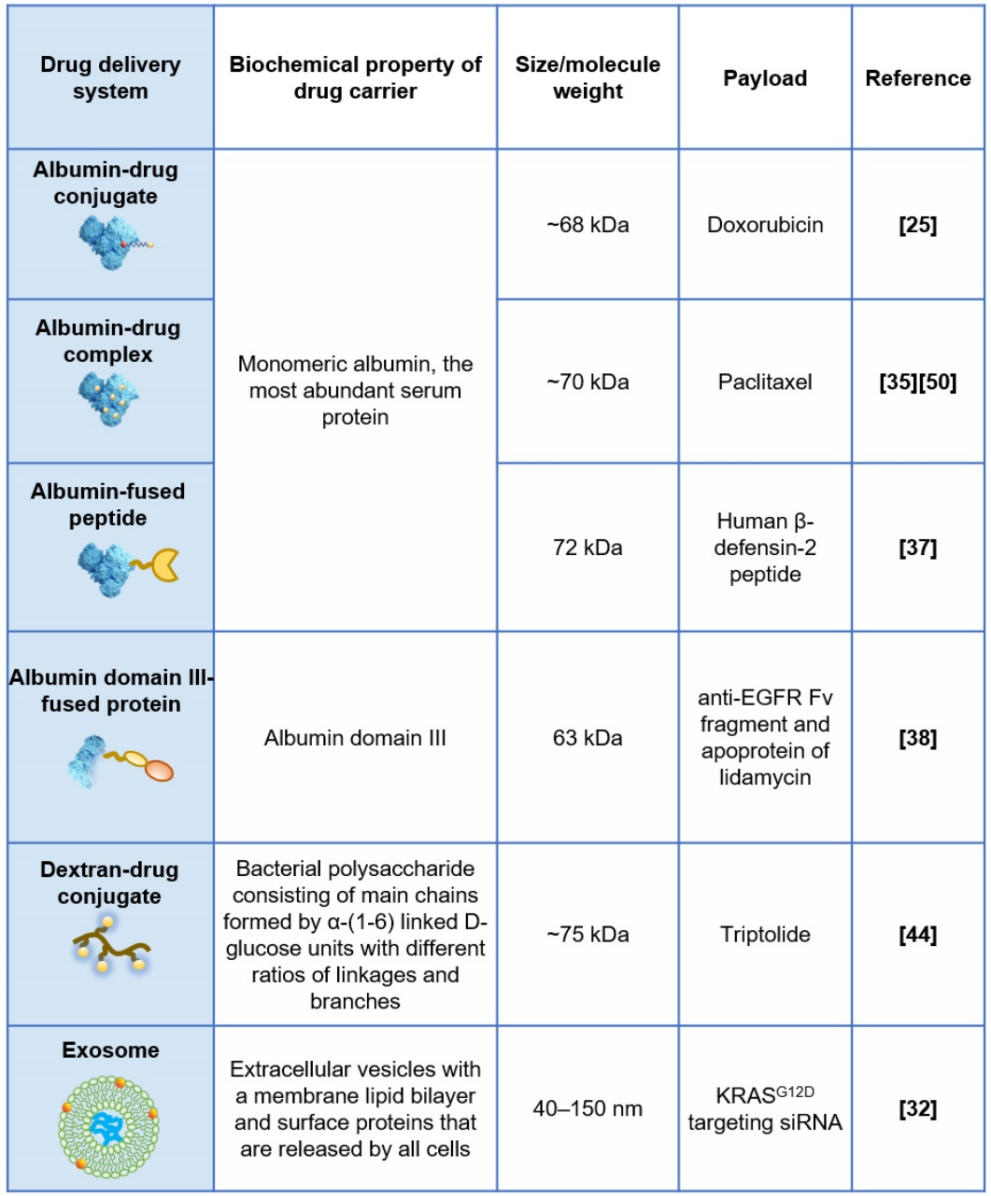
Drug delivery systems exploiting macropinocytosis of KRAS mutant cancer cells.

In summary, a variety of drug delivery systems with different physical and chemical properties have been reported to enter KRAS mutant cancer cells through macropinocytosis (Figure 1). Among them, albumin-based systems have been studied intensively. Despite the fact that most of the published studies confirmed that KRAS enhanced uptake and improved efficacy both *in vitro* and/or *in vivo*, the clinical significance of such a drug delivery strategy has yet to be validated.

## Opportunities that synergize with macropinocytosis to further optimize drug delivery

The entry of complex delivery systems into KRAS tumor cells is only the first step to achieve effective drug delivery, followed by the process of intracellular transport, dissociation and release of the drug payload. Taking the albumin-drug conjugate as an example, after being encapsulated in the macropinosome, the protein and linker need to be degraded in acidic lysosomes to release the drug, and then the free drug reaches the site of function to induce corresponding pharmacological effects. In this dynamic intracellular process, a complex biological signalling network and microenvironment within the involved organelles could contribute to the final pharmacological effects. Consequently, distinct properties of KRAS mutant cancer cells compared to normal cells in these processes could also act in synergy with enhanced macropinocytosis in the pursuit of an ultimate goal for KRAS-targeted drug delivery. For example, the unique properties of lysosomes (pH, composition, abundance and degradation activity of lysosomal hydrolase, etc.) of KRAS mutant tumor cells, if any, could guide us in designing conditional active drug delivery systems. Unfortunately, it appears that there were few conclusive studies in this area. Although enhanced albumin degradation was observed in KRAS mutant tumor cells, as revealed by DQ-BSA (a probe that displays fluorescence after degradation) 16, it is difficult to precisely quantify the contribution of the intrinsic differences in lysosomal function due to the enhanced upstream albumin uptake, as well as the dynamic interaction between macropinosomes and lysosomes.

The neonatal Fc receptor (FcRn), which is responsible for albumin recycling by binding with it in a pH-sensitive manner, shields albumin from lysosomal degradation to achieve a long half-life (~19 days). We previously observed that in pancreatic cancer cells, albumin recycling was decreased while lysosomal catabolism of albumin was increased upon the reduction of FcRn expression, thus sensitized KRAS mutant PDAC to an albumin-conjugated drug but not to an unconjugated drug 25. Although the intriguing connection between KRAS genotype and FcRn expression level remains blurred, the two critical regulators of albumin metabolism have demonstrated synergistic impacts on the sensitivity of pancreatic cancer to albumin-conjugated drugs both *in vitro* and *in vivo*. These results imply an enlarged therapeutic window of albumin-conjugated drugs against PDAC which harbors KRAS mutation and also lacks FcRn expression.

Li and colleagues found that in KRAS mt cells, the macropinocytosis of nab-PTX can be further enhanced therapeutically thus further improving the efficacy of the drug 35. Among 13 tested compounds, six hits were found, including an IGF1R inhibitor AXL1717, which showed the ability to enhance nab-PTX uptake in two independent KRAS mt cancer cell lines 35. *In vitro*, pretreatment with AXL1717 or another IGF1R inhibitor linsitinib decreased the IC_50_ of nab-PTX but not solvent-based PTX in PDAC cells with inducible KRAS^G12D^ by roughly >5-10-fold. *In vivo*, simultaneous dosing of AXL1717 and nab-PTX also notably improved tumor growth inhibition efficacy and extended the survival of allograft animal models. These observations led to the conclusion that an IGF1R-targeted kinase inhibitor enhances nab-PTX macropinocytotic uptake and efficacy, suggesting the potential of combination therapy.

## Limitations in the current qualitative and quantitative methods for macropinocytosis detection

EIPA is the most commonly used tool reagent to qualitatively test whether cell entry of a given drug delivery system is macropinocytosis-dependent. However, to be precise, EIPA is a selective inhibitor of Na^+^/H^+^ exchangers other than a specific macropinocytosis inhibitor, which could indirectly inhibit macropinocytosis by adjusting the cytosolic pH and affect the recruitment and activation of Rac1 and Cdc42 to membrane ruffles. According to other reports as well as our own experiences, treatment with 50-75 μM EIPA for 30 mins could induce nonspecific endocytosis-independent effects and even unignorable cell viability in some sensitive cells.

Therefore, it would be quite helpful to conduct further studies aim to identify the specific regulators to macropinocytosis and develop corresponding inhibitors with precise working mechanisms. In addition to the mechanistic underpinnings of oncogenic KRAS-induced macropinocytosis that have been reviewed elsewhere 12, new mechanistic links between RAS signals and macropinocytosis induction have been gradually revealed 24. Recently, Yao and colleagues revealed that syndecan 1 (SDC1) is a critical mediator for oncogenic RAS-induced macropinocytosis in PDAC using* in vivo* proteomic surfaceome screening 25. Lin et al. attempted to identify novel macropinocytosis inhibitors using an FDA-approved drug library. Among 640 tested compounds, imipramine, which is used for depression treatment, was found to be able to potently inhibit macropinocytosis without exerting cytotoxic effects or inhibiting other endocytic pathways in cancer cells, as well as in dendritic cells and macrophages, whereas the mechanism has not yet been explored 45. However, whether these newly discovered regulatory proteins (such as SDC1) or compounds have sufficient specificity to detect macropinocytosis still needs further research. At present, it is worthwhile to combine multiple methods (i.e., morphological definition, response to amiloride inhibition and growth factor stimulation) to define macropinocytosis.

The most commonly reported macropinocytosis quantification method is the one established by Commisso *et al.*, briefly, first incubate the cells or tissue samples of interest in serum-free medium containing 1-2 mg/mL fluorescently labelled dextran (70 kDa) for 30 minutes, after washing and fixation, then analyze the cells attached on glass slides by fluorescent microscope. Sometimes, FACS is also used as an auxiliary verification method. Jin et al. established a real-time live cell surface imaging method to observe the dynamic macropinocytosis process using three-dimensional-structured illumination microscopy, providing a new approach for qualitative observation. They acquired real-time morphological data of internalized structures (numbers, depth, size of macropinocytic cups) on the surface of MIA PaCa-2 cells during macropinocytosis 46.

In addition, the experimental approaches for the analysis of macropinocytosis in individual patients are also quite limited, which might be a bottleneck in clinical translation for macropinocytosis-exploiting therapeutic strategies. Although some *ex vivo* studies have investigated several freshly acquired human tumor tissues, information regarding the levels and heterogenicity of macropinocytosis in the tumors of patients has not been reported. Moreover, most of the patients with metastatic tumors are not suitable for surgery, limiting the application of the *ex vivo* analytical methods that require isolated tumor tissues. A quantitative, real-time, *in vivo* evaluation method, would be highly desired. On the other hand, identifying reliable biomarkers for elevated macropinocytosis that can be easily detected will be of great value but also faces daunting challenges.

## Off-target risks of exploiting macropinocytosis for targeted drug delivery

**Figure 2:**
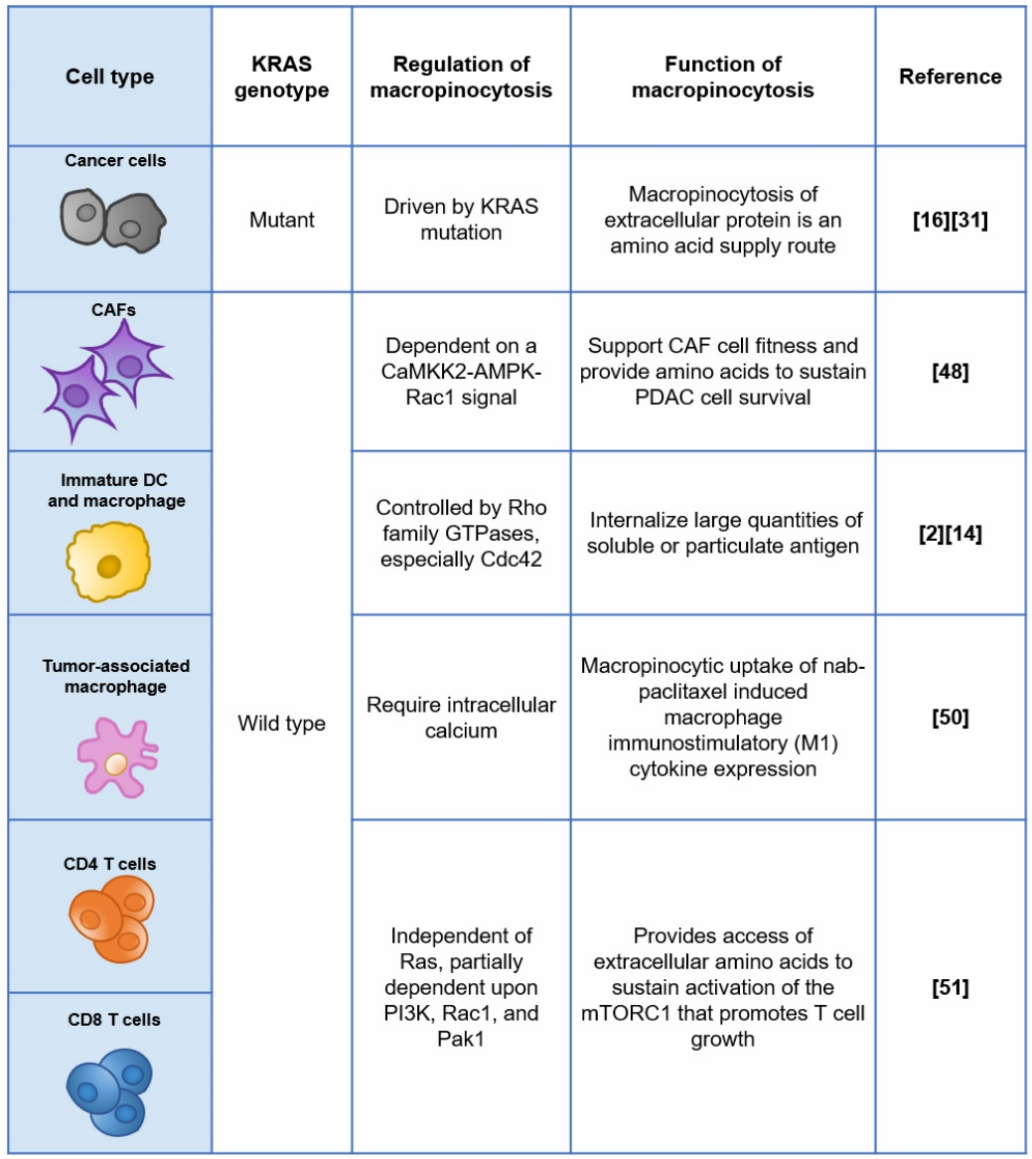
The regulation and functions of macropinocytosis in different cell types.

Off-target risks are critical for the efficacy and safety of macropinocytosis-based drug delivery strategies. Non-specific uptake in healthy tissues such as the liver and spleen, facilitated by a network of phagocytic cells (referred to as the reticular endothelial system, or mononuclear phagocyte system), is an important off-target concern for nanomedicines including macropinocytosis-exploiting drug delivery systems discussed earlier. The effect of physicochemical and surface properties on *in vivo* fate of drug nanocarriers has been intensively reviewed elsewhere 47. For macropinocytosis-based drug delivery strategies, further off-target risk caused by the heterogeneity of macropinocytosis at tissue and cellular levels would be an extra consideration. For instance, beyond RAS-transformed tumor cells, some non-transformed mammalian cells were also reported to have constitutive macropinocytosis, as summarized in Figure 2.

Within the tumor microenvironment (TME) of PDAC, KRAS mutant tumor cells are not the only cell category displaying elevated macropinocytosis, while other non-cancerous cells, such as KRAS wild-type cancer-associated fibroblasts (CAFs), which occupy the majority of the tissue volume, demonstrate similar behavior 48. Zhang and colleagues found that stromal CAFs marked by α-SMA exhibited robust levels of macropinocytosis within KPC-derived orthotopic PDAC tumors, while PDGFR-expressing fibroblasts residing in normal murine pancreas did not display appreciable macropinocytosis. Mechanistically, macropinocytosis in CAFs is not reliant on EGFR signalling and instead is dependent on a CaMKK2-AMPK-Rac1 signal that is potentiated by elevated cytosolic Ca^2+^ 48. Their study highlighted the functional role of macropinocytosis in the tumor stroma to support CAF cell fitness and provide amino acids to sustain PDAC cell survival, suggesting that CAFs could also be targeted by macropinocytosis-aiding drug carriers, and the net effect is unpredictable due to the complicated and contradictory role of CAFs.

Importantly, some immune cells have the capability of constitutive macropinocytosis. For example, immature dendritic cells (DCs) but not mature DCs have a very robust process of constitutive macropinocytosis, which is controlled by the Rho GTPase Cdc42 and Rac-dependent remodelling of the actin cytoskeleton 49. Cullis and colleagues reported that nab-PTX is internalized by macrophages via macropinocytosis predominantly and is capable to drive M1 macrophage polarization *in vitro* and *in vivo* 50. This study, of course, revealed a previously unappreciated mechanism of action of nab-PTX but also suggested that nab-PTX may induce off-target drug delivery into macrophages with active macropinocytosis. More recently, the active uptake of macropinocytosis probes (70 kDa dextran or albumin) was observed in both murine and human CD4^+^ and CD8^+^ T cells, and the T cells activated upon CD3/28 monoclonal antibody stimulation increased the extent of probe uptake 51. Mechanistically, macropinocytosis could promote T cell growth by providing access to extracellular amino acids that are essential for the sustained activation of the mechanistic target of rapamycin complex 1 (mTORC1), even under amino acid replete conditions, as reported 51. With the great breakthrough of immunotherapy in the treatment of cancer, we now have an unprecedented realization of the great significance and energy of the immune system in resisting tumors. A well-designed delivery system that enters KRAS mutant cancer cells through macropinocytosis and kills them efficiently while sparing immune cells will be much more promising.

## Pitfalls in our current understanding of macropinocytosis

Last but not least, there are some widely conceived beliefs in this field, which have not yet been rigorously proven. First, macropinocytosis is a nonselective, bulk uptake process. Second, 70 kDa dextran is a marker of macropinocytosis. These two notions are contradictory: with the belief that macropinocytosis is a bulk uptake process without substrate selectivity, we are puzzled facing this question: when all the options are listed on the menu, do gourmets with KRAS mutations take on all these substrates, such as various components of the TME including extracellular proteins, drug delivery systems with different physical and chemical properties and different drug encapsulation mechanisms etc., with the same mechanisms and kinetics yet without any preference?

Intuitively, the answer will be “not very likely”. There are a few pieces of evidence worth discussing. First, the size range of the substances that enter KRAS mt cancer cells via macropinocytosis was not the maximum value of the theoretical volume of the macropinosome structures. Jin et al. observed that single-walled 2 μm carbon nanotubes and 420 nm SiO_2_ nanoparticles could not be internalized into MIA PaCa-2 cells, although their sizes were covered in the size range of macropinosomes 46. Second, fluorescent dextran with different molecular weights has distinct endocytic pathways. Li et al. assessed the dependence of basal uptake of dextran with distinct molecular weights on macropinocytosis by using the Na^+^/H^+^ exchange-inhibitor amiloride and found that macropinocytosis dominates the fluid uptake of 70 kDa dextran, but not 10 kDa dextran, into HeLa cells 52. The uptake of 70 kDa dextran was found to be inhibited to a much larger extent (up to 76%) than that of 10 kDa dextran (32%). Interestingly, although their molecular weights differ by 7 times, their hydrodynamic sizes differ by only 2-3 times, and the mechanism of how this difference affects macropinocytosis is still unknown.

These studies demonstrated that the physical and chemical properties of the substrate, size and molecular weight may all affect its internalization by macropinocytosis mechanism. At this stage, all we have seen is merely the tip of the iceberg regarding the science of macropinocytosis biology, and the presumption that it is a “non-selective” endocytosis process might just be an oversimplified understanding.

## Concluding remarks

Multiple independent studies have demonstrated that tumor cells with endogenous KRAS mutations have enhanced macropinocytosis, which is oncogenic KRAS-dependent. These cells take in extracellular proteins through macropinocytosis and degrade them into amino acids, providing sources for the biosynthesis of tumor cells. In addition to extracellular proteins, many drug carriers can also enter KRAS mutant tumor cells through macropinocytosis, encouraging continuous attempts exploiting macropinocytosis to deliver drugs into tumor cells with KRAS mutations, and promising results (elevated uptake and improved efficacy) have been observed *in vitro* and *in vivo*. However, we still have some unknowns and risks: the limitation of specific chemical and genetic interference tools makes it tricky to identify macropinocytosis; the lack of real-time, quantitative methods for macropinocytosis level detection *in vivo* is an obvious blockage in translational medicine research; persistent macropinocytosis of macrophages and dendritic cells suggests the risk of off-target effects, etc*.* Thinking about these tough questions and seeking solutions by well-designed studies will truly help us understand macropinocytosis and thus guide us in the design of drug delivery systems, paving the way for the clinical application of this drug delivery strategy in the future.

## Figures and Tables

**Table 1:** Relative macropinocytosis activity of KRAS mt cancer cells compared to KRAS wt cells

References	Macropinocytosis assay	Sample system	Relative levels
Commisso et al., 2013 16	TMR-dextran was added to the serum-free medium at a final concentration of 1 mg/mL for 30 min at 37 °C. Total fluorescent particle area per cell was determined from at least five fields using the ImageJ.	MIA PaCa-2 (KRAS^G12C^) compared to BxPC-3 cells	8-fold
		KRAS^G12V^ NIH 3T3 cells compared to untransformed control cells	4-fold
Qian et al., 2014 27	Macropinocytosis assays were performed as described previously. 16	A549 cells (KRAS^G12S^) vs. MCF7 breast cancer cells	~100 fold
Kamphorst et al., 2015 31	Freshly acquired human tumor specimens (n=5) were incubated with high molecular weight TMR-dextran (1-2 mg/mL, at 37 °C for 20-30 minutes) and intracellular uptake of TMR-dextran was assessed by fluorescence microscopy.	CK19-positive tumor cells vs. normal adjacent tissue	An accurate quantitative comparison is not provided
Kamerkar et al. 2017. 32	Macropinocytosis assays were performed as described previously. 16	PANC-1(KRAS^G12D^) and BxPC-3	~9 fold
Aaron Hobbs et al., 2020 20	Macropinocytosis assays were performed as described previously. 16	KRAS^G12D^-transformed RIE-1 cells compared to untransformed control cells	>40 fold
		KRAS^G12V^-transformed RIE-1 cells compared to untransformed control cells	~40 fold
Aubert et al., 2020 33	Cells were incubated in serum-free media containing 0.5 mg/mL of Lysine-fixable TMR-Dextran (10 kDa) for 30 min at 37 °C.	Isogenic intestinal epithelial cell model (IEC-6) stably expressing KRAS^G12V^ compared to untransformed control cells	~3 fold
Liu et al., 2019 25	Cells were seeded into 12 wells plates (5 × 10^5^ cells/well) and pulsed for 0, 1, 2, 4, or 8 h with 0.2-5 mg/mL 70 kDa FITC-dextran at 37 °C in complete growth medium.	BxPC-3 stably expressing KRAS^G12V^ compared to BxPC-3 control cells	1-3 fold

## Abbreviations

NHE: Na^+^/H^+^ exchanger
PDAC: pancreatic duct adenocarcinoma
TME: tumor microenvironment
CAFs: cancer-associated fibroblasts
KPC: Pdx1-Cre, LSL-Kras^G12D/+^, LSL-p53^R172H/+^
α-SMA: α-smooth muscle actin
EIPA: 5-(NEthyl-N-isopropyl) amiloride
Cdc42: cell division control protein 42
FACS: fluorescence-activated cell sorting
wt: wild type
mt: mutant
HAS: human serum albumin
nab-PTX: albumin-bound formulation of paclitaxel
NSCLC: non-small cell lung cancer
FcRn: neonatal Fc receptor
DCs: dendritic cells
mTORC1: mechanistic target of rapamycin complex 1
